# Effects of Vitamin D Supplementation during the Induction and Progression of Osteoarthritis in a Rat Model

**DOI:** 10.1155/2012/156563

**Published:** 2012-10-14

**Authors:** E. C. Castillo, M. A. Hernandez-Cueto, M. A. Vega-Lopez, C. Lavalle, J. B. Kouri, V. Ortiz-Navarrete

**Affiliations:** ^1^Departamento de Infectómica y Patogénesis Molecular, Centro de Investigación y de Estudios Avanzados (CINVESTAV), IPN, Avenida IPN No. 2508, 07360 México, DF, Mexico; ^2^Departamento de Biomedicina Molecular, Centro de Investigación y de Estudios Avanzados (CINVESTAV), IPN, Avenida IPN No. 2508, 07360 México, DF, Mexico; ^3^Unidad de Investigación en Infectología e Inmunología, Hospital de Infectología Dr. Daniel Méndez Hernández, Centro Médico Nacional La Raza, IMSS, Avenida Jacarandas s/n, 02990 México, DF, Mexico; ^4^Facultad de Medicina, Universidad Nacional Autónoma de México (UNAM), Circuito Escolar s/n, 04510 México, DF, Mexico

## Abstract

Epidemiological studies correlate low levels of vitamin D with the osteoarthritis (OA) progression. Cytokines and metalloproteases play a major role in OA promoting the inflammation and degradation of the cartilage and can be induced through the Toll-like receptor (TLR) pathway. The aim of this study was to evaluate the protective effect of vitamin D supplementation on the development of osteoarthritis (OA) through examining the genetic regulation of TLRs, cytokines, and metalloproteases in chondrocytes as well as the wideness of cartilage in rats with OA. Our results demonstrate that the signaling through TLR-4 is a proinflammatory mechanism in osteoarthritis that drives the upregulation of MMP-3, IL-1**β**, and TNF-**α** gene expression, leading to cartilage degradation and inflammation. Vitamin D supplementation had a protective effect during the onset but not during the chronic stage of OA in the rat model.

## 1. Introduction

It has been shown that TNF-*α* synthesis decreases in monocytes treated with vitamin D (1,25(OH)_2_D_3_) due to the downregulation of TLR-2 and TLR-4 signaling [1]. In this context, epidemiological studies and experimentally induced animal models suggest that the status of vitamin D is associated with the severity of inflammatory diseases such as rheumatoid arthritis [2] and OA [3]. In particular, vitamin D deficiency has been positively associated with knee [4] and hip [5], OA progression [2, 6], cartilage loss [7], and low bone mineral density [3]. However, it has also been shown that vitamin D deficiency is associated with only certain age groups [4, 6, 8] and with certain factors, such as pain, rather than with radiographic changes [9, 10], while other studies have found no association between vitamin D and OA [8, 11, 12], even in patients diagnosed with OA who were using vitamin D supplements [13]. 

Because epidemiological studies can only evaluate the effect of vitamin D in certain stages of OA, we aimed to define whether vitamin D has a protective effect during the onset or progression of OA using a rat model. The effect of vitamin D was evaluated by measuring condyle wideness and the gene expression of key cytokines, MMPs, and TLRs that are involved in the development of OA.

## 2. Methods

### 2.1. Rats and Cartilage Samples

Male Wistar rats weighing 130–150 g were kept in acrylic cages with a stainless steel cover. The rats were housed in a controlled environment with a 12 h light/dark cycle, constant temperature (22°C) and humidity (70%), and free access to food and water. Their diet was standard chow (Formulab Diet 5008) containing 1% calcium, 0.65% phosphorous, and 3.3 IU/g of vitamin D_3_.

**Figure 1:**
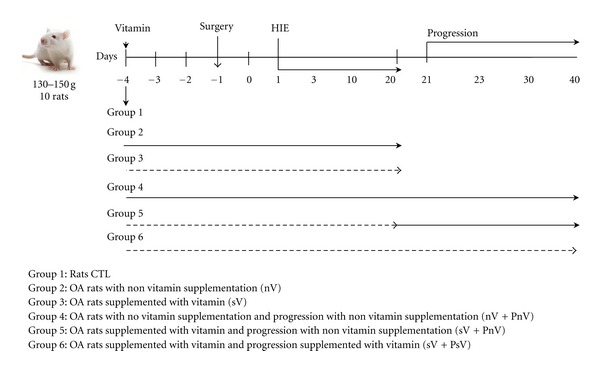
Experimental groups. A schematic example of the 20-day induction group and the 40-day progression group of rats with OA with or without supplementation is shown. The same scheme was applied for the other induction (3 and 10 days) and progression (20 and 40 days) groups. Vitamin D (4 IU/kg/d) was administrated orally with an esophagogastric cannula 14 HALO. The arrow indicates the group without vitamin supplementation (nV); the pointed arrow indicates the supplementation with vitamin D (sV). Each group was conformed by 10 rats, and every experiment was performed at least by triplicate.

Cartilage samples were obtained from 10 knees of healthy (control (CTL)) or OA rats per experimental group. The local ethics committee of the UPEAL, CINVESTAV-IPN (NOM-062-ZOO-1999), approved this study. The rats were anesthetized with a ketamine 60 mg/kg and xylazine 4 mg/kg solution, and OA was induced in the knee of the right hind leg by a partial meniscectomy, which is the transecting of the medial collateral ligament (MCL) [14], and high-impact exercise (HIE) for 1, 3, 6, 8, 10, or 20 days. For the progression groups, the rats exercised for 3, 10, or 20 days and then were maintained for 20 additional days without exercise (Figure 1), resulting, study periods of 23, 30, and 40 days, respectively. Explants were obtained from the femoral condyles and tibial plateau cartilage from joints where the partial meniscectomy was performed.

The high-impact exercises began two days after surgery. The rats were forced to slide from side to side for 2 minutes in a box, and then the rats were dropped continuously for another 2 minutes. Finally, the rats were forced to jump as the box was continually shaken vertically for 1 minute. This exercise cycle was repeated 3 times each day.

### 2.2. Vitamin D Administration

Capsules of vitamin D (1,25-dihydroxyvitamin D_3_) from Member's Marck (Bentonville, AR) were dissolved in corn oil (vehicle). The doses were 100 ng (4 IU), 1 *μ*g (40 IU), 10 *μ*g (400 IU), and 100 *μ*g/kg/day (4000 IU) and were administrated orally with an esophagogastric cannula 14 h after lights on (14HALO [15]). Each dose was administered 3 days before surgery, and daily administration continued until the last day of OA induction. For the evaluation of 4 IU of vitamin D supplementation during OA progression, three experimental subgroups were included: (1) rats without vitamin supplementation during HIE + progression without vitamin supplementation (nV + PnV), (2) rats supplemented with the vitamin during HIE + progression without vitamin supplementation of vitamin (sV + PnV), and (3) rats supplemented with the vitamin during HIE + progression with vitamin supplementation (sV + PsV) (Figure 1).

### 2.3. RNA Isolation, cDNA Synthesis, and Real-Time Quantitative Polymerase Chain Reaction (PCR) Amplification

The cartilage samples were enzymatically digested, and the total RNA was isolated from chondrocytes for real-time reverse transcriptase PCR analysis (qPCR) of TLR-1, TLR-2, TLR-4, TLR-6, IL-1*β*, TNF-*α*, IL-6, MMP-3, MMP-9, and MMP-13 (Real-Time PCR System, model 7500, Applied Biosystems, Carlsbad, CA, USA) using the TRIzol LS Reagent (Invitrogen, USA). The first strand of cDNA was synthesized from 1.0 *μ*g total RNA using a high capacity cDNA reverse transcription kit with RNase inhibitor (Applied Biosystems, Carlsbad, CA, USA). Glyceraldehyde 3-phosphate dehydrogenase (GAPDH) mRNA was used as an endogenous control to allow for the relative quantification of the genes of interest. The qRT-PCR was performed with the fast SyBR-Green master mix (Applied Biosystems, Carlsbad, CA, USA) on both the targets and the endogenous control (the probes used are shown in Table 1). The amplified PCR products were quantified by measuring the calculated cycle thresholds (C_T_). The amounts of specific mRNA in the samples were calculated by the ΔΔC_T_ method. The mean value of the CTL chondrocytes target levels was used as the calibrator (one per sample), and the results were expressed as the *n*-fold difference relative to normal controls for the relative expression level data (2^−ΔΔC_T_^).

The quality of the samples was determined by the difference in the absorbance at 260 and 280 nm. The RNA had an average A260/A280 ratio of 1.87. The RNA quality was also assessed by the electrophoresis of the total RNA followed by staining with ethidium bromide. In all samples, bands of 5 kb and 2 kb, corresponding to the 28S and 18S rRNAs, were observed. The cDNA obtained had an A260/A280 average value of 1.86.

### 2.4. Histological Analysis

Cartilage samples were obtained from 2 knees of healthy (control; CTL) or 20 days OA rats with or without vitamin D supplementation. Samples from each group were fixed with 4% formol, embedded in paraffin, cut at a thickness of 4 *μ*m and stained with hematoxylin and eosin (HE).

### 2.5. Statistical Analysis

The data are shown as the mean ± the standard error (standard error of the mean; SEM). The statistical significance is indicated on the graphs as follows: *P* < 0.05 (*), *P* < 0.01 (**), or *P* < 0.001 (***), with a 95% CI. The expression levels of TLRs, MMPs, and cytokines were measured in five independent experiments (*n* = 5) for the OA kinetic analysis and in three independent experiments (*n* = 3) for the vitamin D dose-response and the OA progression analysis. The statistical analyses were performed by the Graph Pad Instat program (Graph Pad Software Inc., San Diego, CA, USA). A two-way analysis of variance (ANOVA) followed by Tukey's post hoc test was performed to compare means between the experimental groups without vitamin supplementation and the CTL group or a test for linear trend when applicable. A one-way ANOVA analysis using Dunnet's post hoc test was performed to compare the different doses of vitamin D. Student's *t*-test was used to compare between the progression groups without vitamin supplementation and the progression group supplemented with vitamin D since the induction. Finally, the correlation analysis was performed using Pearson's correlation.

## 3. Results

### 3.1. Expression of TLRs, Proinflammatory Cytokines, and MMP Genes in Chondrocytes during OA Induction

**Figure 2:**
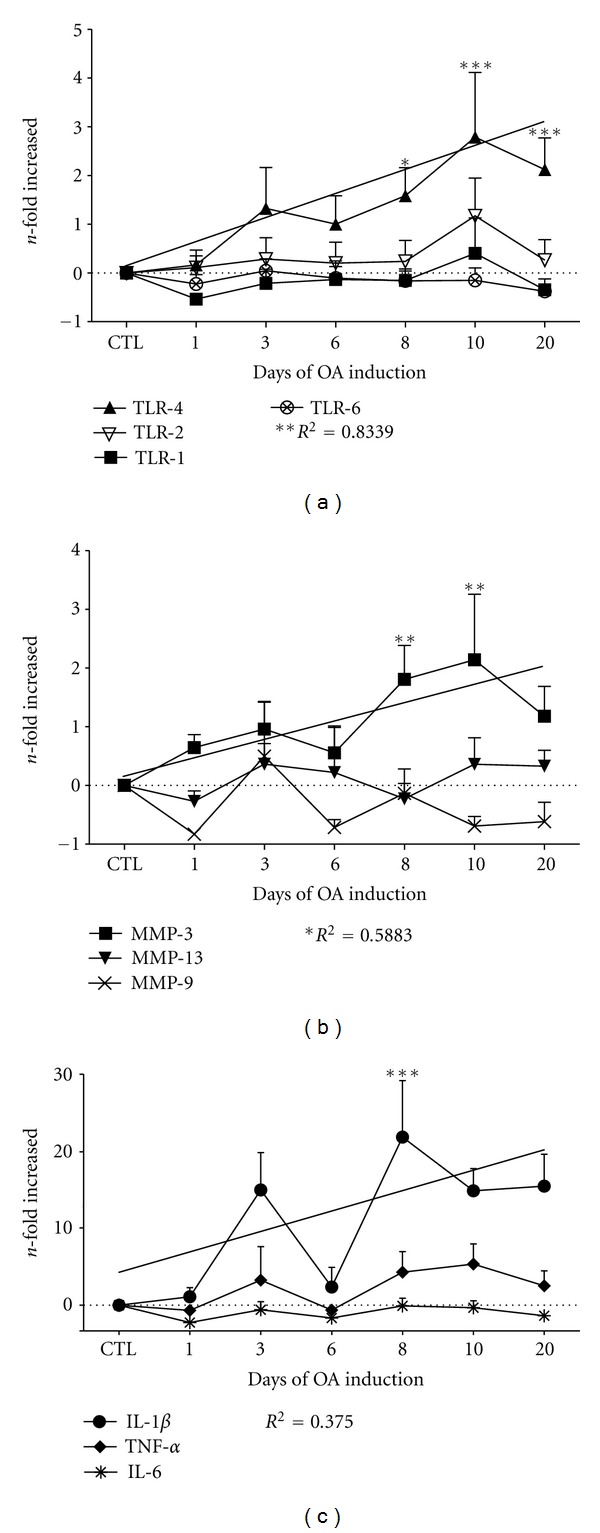
TLR-4, MMP-3, IL-1*β*, and TNF-*α* expression was induced during osteoarthritis induction. Chondrocytes were collected and assayed for gene expression by real-time RT-PCR. The relative expression levels were normalized to the expression of the housekeeping GAPDH gene expression and presented as the *n*-fold difference. (a) TLR-2 and TLR-1 showed a similar pattern of expression during OA induction, whereas TLR-6 was only expressed during the acute stage of OA. TLR-4 showed a remarkable increase in expression with respect to the CTL group throughout OA induction, with a significant linear trend (*R* ^2^ = 0.8339; *P* = 0.0041). (b) The relative expression levels of MMP-3, MMP-9, and MMP-13, normalized to GAPDH gene expression, are shown. MMP-3 expression increased during OA induction (*R* ^2^ = 0.5883, *P* = 0.0219) and was highest on days 8 (1.8-fold; 95% CI 0.005241 to 3.609) and 10 (2.1-fold; 95% CI 0.3375 to 3.941). (c) The relative expression of IL-1*β*, IL-6, and TNF-*α*, normalized to GAPDH gene expression, is shown. IL-1*β* gene expression increased during OA induction (*R* ^2^ = 0.375), while TNF-*α* gene expression changed modestly (*R* ^2^ = 0.4173), with the highest increase on day 10 (1.6-fold). The results are pooled data from five independent experiments with 50 total rats. The asterisks indicate *P < 0.05, **P < 0.01, and ***P < 0.001, which were determined using a 2-way ANOVA followed by Tukey's post hoc test to compare the experimental groups with the CTL group. The error bars indicate the SEM.

To evaluate TLR-1, TLR-2, TLR-4, and TLR-6 gene expression during OA development in this rat model, we isolated cartilage chondrocytes from rats subjected to 1, 3, 6, 8, 10, and 20 days of OA induction and assessed TLR mRNA levels using quantitative RT-PCR.  Figure 2(a) shows that TLR-1, TLR-2, and TLR-6 were expressed in the chondrocytes throughout the OA induction with no differences in expression levels compared to the control group. Meanwhile, an increase in the level of TLR-4 mRNA expression was observed (linear trend, *P* = 0.0041; *R* ^2^ = 0.8339) from day 8 (1.6-fold; *P* < 0.05) to 20 (2.1-fold; *P* < 0.001) with maximal expression on day 10 (2.8-fold; *P* < 0.001).

TLR-4 activation results in the expression of molecules associated with damage, such as MMPs and inflammatory cytokines. Therefore, we evaluated the mRNA expression of MMP-3, MMP-9, MMP-13, IL-6, TNF-*α*, and IL-1*β*. The expression of MMP-9 and MMP-13 was detected during the progression of OA, but there were no major changes in their expression between the OA and the CTL groups (Figure 2(b)). In contrast, MMP-3 expression was higher in the OA groups on days 8 (1.8-fold; *P* < 0.01) and 10 (2.1-fold; *P* < 0.01), with a linear trend suggesting an increase in expression (*R* ^2^ = 0.5883; *P* = 0.0219), as shown in Figure 2(b). Similar to MMP-9 and MMP-13 expression, the IL-6 expression level remained similar to that of CTL, while the expression level of TNF-*α* (*R* ^2^ = 0.4173) and IL-1*β* (*R* ^2^ = 0.375) was elevated throughout OA induction (Figure 2(c)). There was a peak in the IL-1*β* expression on day 8 (18-fold; *P* < 0.001), suggesting that this cytokine plays an important role during the onset of OA pathogenesis.

TLR-4 expression correlated with the expression of MMP-3 (*R* ^2^ = 0.7692; *P* = 0.0095), TNF-*α* (*R* ^2^ = 0.7127; *P* = 0.0169), and IL-1*β* (*R* ^2^ = 0.3954; *P* = 0.1304), as shown in Supplementary Figure 1, (see Supplementary Material available online at doi:10.1155/2012/156563). These results suggest that the activation of the TLR-4 pathway plays an important role in OA pathogenesis by inducing MMP-3, TNF-*α*, and IL-1*β* expression. However, IL-1*β* expression may not be entirely dependent on TLR-4 because it also depends on the NOD-like receptors pathway [16].

### 3.2. Effect of Vitamin D on OA Cartilage (Dose Response)

**Figure 3:**
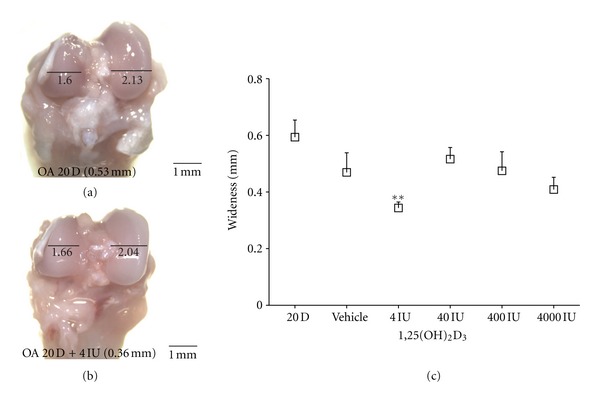
Vitamin D reduces the wideness of OA condyles. (a) A representative image of the femoral condyles from a rat with 20 days of OA without treatment. (b) A representative image of the femoral condyles from a rat treated with 4 IU/kg/day of vitamin D. The difference in wideness was taken as an indicator of the degree of severity of OA hypertrophy. (c) The graph presents the average wideness at a given dose from 3 different experiments with 15 total rats. A decrease in the severity of 0.34 ± 0.047 mm (95% CI 0.05367 to 0.4459, **P < 0.01) was determined using a one-way ANOVA with Dunnet's post hoc test to compare all of the conditions with the control group (OA of 20 days without vitamin D). The error bars indicate the SEM. The scale bar = 1 mm 8x.

If the activation of TLRs and the synthesis of cytokines and MMPs are associated with OA and if vitamin D modulates TLR-4 activation, then there should be detectable changes in the joint in response to such modulation. Some of the main characteristics of OA are joint hypertrophy and cartilage and bone erosion, which are associated with increased fibrotic tissue at the borders of the joint. For that reason, we next evaluated the effect of supplementation with different vitamin D doses (4, 40, 400, and 4000 IU/kg/d) during the induction of OA for 20 days (OA20). We measured the OA condyles and nonmeniscected condyles (right and left, resp.), and the difference between them was considered an indicative value of OA hypertrophy severity (Figures 3(a) and 3(b)). The wideness of the condyles in OA20 rats without vitamin D was used as the control (Figure 3(a)). We observed that the 4 IU/kg/day dose was the only dose that caused a reduction in the wideness of the OA condyles (*P* < 0.01; 0.34 ± 0.047 mm) (Figure 3(c)).

### 3.3. Effect of Vitamin D Supplementation on OA Induction

**Figure 4:**
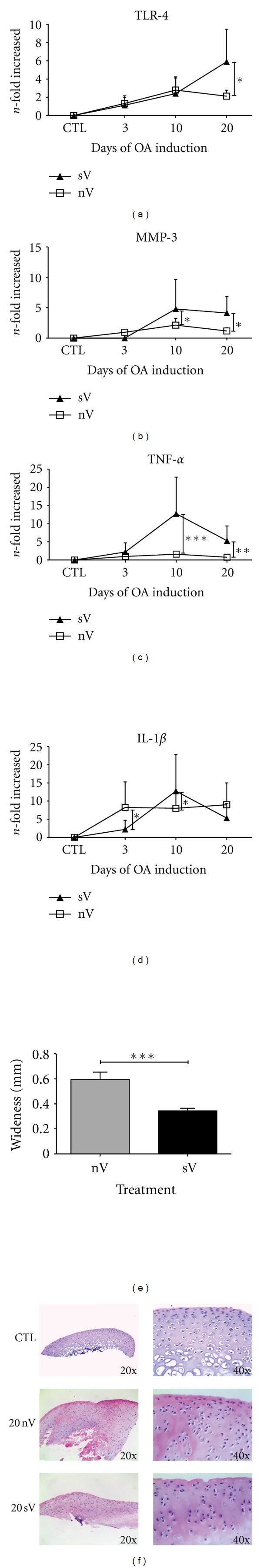
The protective effect of vitamin D is independent of the transcriptional modulation of TLR-4. The evaluation was performed during the OA induction period for (a)–(d). The gene expression of TLR4 (a), MMP-3 (b), TNF-*α* (c), and IL-1*β* (d) from rats in the nV group (□) compared with the sV group (▲) is shown. The gene expression levels were normalized to GAPDH gene expression and presented as the *n*-fold difference in expression. (e) A comparison of the hypertrophy (wideness) of the condyles from rats with 20 days of OA that were supplemented with vitamin D (sV) to that of rats with no supplementation (nV). (f) A morphological comparison between condyles of control rats and rats with 20 days of OA induction treated with vitamin D (sV) and nontreated (nV) by HE stain. The supplementation was performed daily with 4 IU of vitamin D. The results are expressed as pooled data from three independent experiments with 16 to 18 total rats (a–d) or with 6 rats per group (e). The error bars indicate the SEM. A *t*-test with an *F* test was performed to obtain the indicated statistical significance values: ^∗^ *P* < 0.05, **P < 0.01, and ***P < 0.001. Abbreviations indicate the following treatments: nV (without vitamin supplementation); sV (with vitamin supplementation).

To assess whether the protective effect of supplementation with 4 IU of vitamin D involves TLR-4 modulation, we first evaluated the expression of TLR4, IL-1*β*, TNF-*α*, and MMP-3 during different stages of OA induction. We established day 3 of induction as the acute stage, day 10 as the intermediate stage, and day 20 as the chronic stage. This classification was established taking in consideration the previous results [14]. TLR-4 expression was upregulated (5.89-fold; *P* = 0.0102  *F* test) until the chronic stage (Figure 4(a)), while MMP-3 expression was upregulated from the intermediate stage (4.79-fold; *P* = 0.0246  *F* test) to the chronic stage (4.13-fold; *P* = 0.0116  *F* test, Figure 4(b)). TNF-*α* was also upregulated during the intermediate and chronic stages (10 days: 12.75-fold; *P* = 0.0004 and 20 days: 5.31-fold; *P* = 0.0041  *F* test), as shown in Figure 4(c). Meanwhile, IL-1*β* expression was downregulated in the acute stage (2.21-fold; *P* = 0.0104  *F* test), which was followed by an upregulation on day 10 (12.75-fold; *P* = 0.0473  *F* test). Finally, IL-1*β* showed the same levels of expression in the chronic stage (5.31-fold; *P* = 0.2422  *F* test) as the nV group (Figure 4(d)). These data suggested that the protective effect (*P* = 0.0010  *t* test) of vitamin D supplementation observed during OA induction (Figures 4(e) and 4(f) and Supplementary Figure 2) is not due to the modulation of TLR-4 activation at the transcriptional level. In Figure 4(f), we can observe after 20 days of OA induction a severe damage with intense hypertrophy and hyperplasia of the cartilage, which diminished with vitamin D supplementation.

### 3.4. Effect of Vitamin D Supplementation on Gene Expression during OA Progression

**Figure 5:**
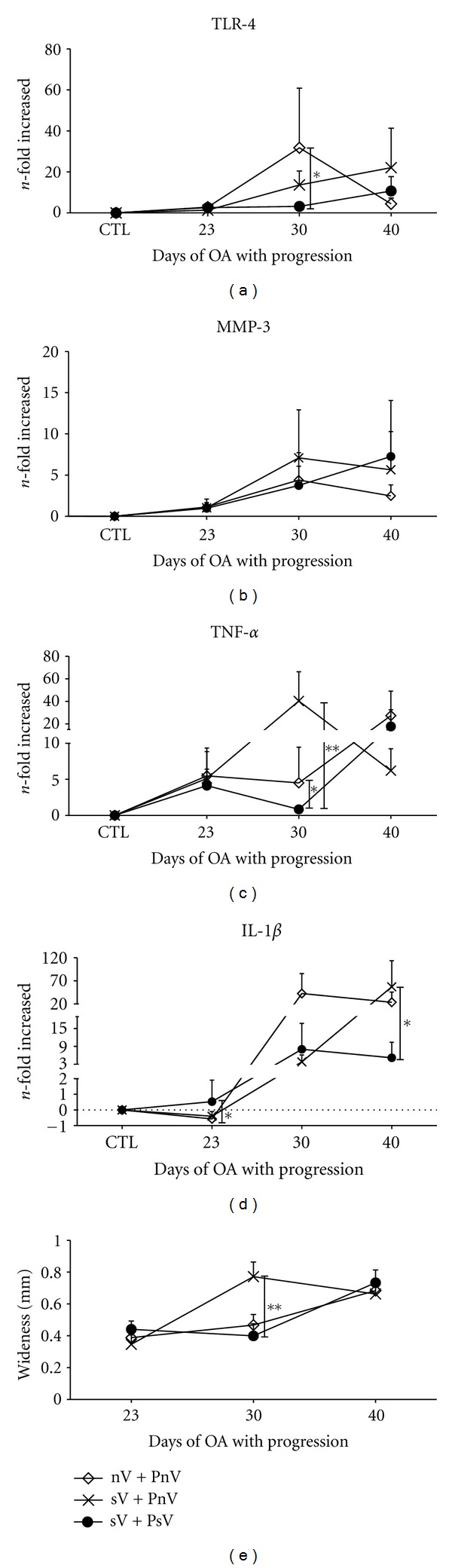
Vitamin D has no effect on OA severity. The evaluation of OA was performed during the OA progression phase.(a)–(d). The gene expression of TLR-4 (a), MMP-3 (b), TNF-*α* (c), and IL-1*β* (d) from rats in the sV + Psv group (●) compared with the sV + PnV (×) and the nV + PnV (*⋄*) groups. The gene expression levels were normalized to GAPDH gene expression and are presented as the *n*-fold difference in expression. (e) A comparison of the hypertrophy (wideness) of condyles from the rats of the sV + PsV (●), sV + PnV (×) and nV + PnV (*⋄*) groups is shown. The results shown are pooled data from three independent experiments with 16 to 18 total rats (a–d) or with 12 to 16 total rats (e). The error bars indicate the SEM. A *t*-test with an *F* test was performed; the notations indicate *P < 0.05 and **P < 0.01. The following groups were included: sV + PsV (vitamin supplementation during induction and progression with supplementation of vitamin); sV + PnV (vitamin supplementation during induction and progression without vitamin supplementation); nV + PnV (without vitamin supplementation during induction and progression without vitamin supplementation). For the supplement, 4 IU of vitamin D was administrated daily.

To mimic the natural progression of OA, we evaluated the effect of vitamin D supplementation in rats with OA progression, as described in Figure 1. The expression of TLR-4 was downregulated at day 30 (28.61-fold; *P* = 0.0125  *F* test) in the sV + PsV group compared to the nV + PnV group (Figure 5(a)). MMP-3 expression was similar between the evaluated groups (Figure 5(b)); while, TNF-*α* expression was downregulated in the sV + PsV group on day 30 compared to the groups: sV + PnV (39.55-fold; *P* = 0.0042  *F* test) and nV + PnV (3.66-fold; *P* = 0.0244  *F* test) (Figure 5(c)). In addition, IL-1*β* was downregulated on day 40 (51.97-fold; *P* = 0.0168  *F* test) in the sV + PsV group compared to the sV + PnV group (Figure 5(d)) and upregulated on day 23 (1.09-fold; *P* = 0.0491  *F* test) compared to the nV + PnV group (Figure 5(d)).

In contrast to the protective effect of vitamin D observed during the OA induction, we did not find any evidence for a protective effect of vitamin D supplementation during OA progression. As is shown in Figure 5(e) and Supplementary Figure 2, the rats of all groups had the same degree of damage from OA at the end of the experiment. In this case, the damage was correlated with the changes observed in the gene expression of TLR-4, MMP-3, and TNF-*α* (Figure 5).

## 4. Discussion

The present study shows the involvement of the TLR-4 pathway in the expression of MMP-3, IL-1*β*, and TNF-*α* during the development of OA. It also shows that vitamin D supplementation has a protective effect on condyle wideness during the induction of OA despite an increase in the expression of the above genes. In contrast, during OA progression, vitamin D supplementation induced the downregulation of TLR-4, IL-1*β*, and TNF-*α* on day 30, but this had no beneficial effect on the disease outcome.

Although human articular chondrocytes express mRNAs for all TLRs (TLRs 1-9) [17] only TLR-2 and TLR-4 expression increased in OA chondrocytes. OA chondrocytes stimulated *in vitro* with ligands for TLR-2 or IL-1*β* induced the release of PGE_2_, MMPs (-1, -3, and -13), and nitric oxide as a consequence of this stimulation [18–20]. Similarly, the stimulation of TLR-4 expression in normal chondrocytes [17, 21], induces the secretion of TNF-*α*, IL-1*β*, MMP-13, and inducible nitric oxide synthase [22]. In this study, we found that TLR-4 was upregulated during OA induction *in vivo* and that its expression correlates with TNF-*α*, IL-1*β*, and MMP-3 gene expression. These data suggest that TLR-4 signaling is important in the pathophysiological response that causes the inflammation and degradation of the cartilage in OA.

The TLR activation pathway induces the expression of the vitamin D receptor (VDR) and CYP27B1 (1*α*-hydroxylase), the enzyme that converts 25-hydroxycholecalciferol into 1,25(OH)_2_D_3_, thereby promoting innate immunity through VDR activation [23, 24]. It has been reported that p65, a subunit of NF-*κ*B, interacts directly with the VDR and that this interaction modulates the VDR and TLR responses [25–27]. The outcome of this TLR-VDR transcriptional partnership is the regulation of the target genes of both receptors. For this reason, we decided to evaluate if the suggested protective effect of vitamin D supplementation on OA was due to a modulation of TLRs, cytokines, and MMPs at the transcriptional level [1] or if vitamin D was in fact associated with more severe OA because VDR expression has also been associated with MMP-3 expression in OA cartilage [28] and the upregulation of inflammatory cytokines [29]. In this study, we observed that vitamin D supplementation differentially modulated the gene expression of TLR-4, TNF-*α*, IL-1*β*, and MMP-3 during OA induction and progression. Vitamin D supplementation induced TLR-4 expression during OA induction but downregulated it during OA progression. These antagonistic responses might be due to the continued mechanical overload that promotes the activation of the inflammatory pathway during OA induction.

This differential response has been observed after inflammatory cytokines modulation. Vitamin D downregulates IL-1*β* and TNF-*α* expression in peripheral blood mononuclear cells via TLR-4 downregulation [22]. In contrast, in human monocyte-derived macrophages activated with lipopolysaccharide, vitamin D stimulates the production of IL-1*β* [28] and downregulates TNF-*α* expression [1, 22, 30]. In our results, we observed both positive and negative effects on cytokines. IL-1*β* increased on day 10 of OA induction (sV) and was downregulated on day 40 of OA progression (sV+PsV). Similar effects on TNF-*α* expression were observed.

Enhancing and suppressive roles for vitamin D have also been described for MMPs expression. Vitamin D has been associated with MMP-3 expression in human OA cartilage. *In vitro* studies show that vitamin D induces the production of MMP-1, MMP-3, and PGE_2_ in chondrocytes stimulated with IL-1*β*, whereas it reduces MMP-1, MMP-3, and PGE_2_ induction by IL-1*β* in rheumatoid synovial fibroblasts [28, 31], which are two scenarios that could be present and contribute to the final state of the cartilage during OA development. Our *in vivo* results agree with the effect described for MMP-3 in chondrocytes *in vitro* with regard to the groups of OA induction and at day 40 of OA progression. However, despite that at day 40, MMP-3 expression was upregulated in OA rats, there were no major changes in the other groups. These data suggest that the effect of vitamin D depends not only on the cell type but also on the activation state of the cells and the inflammatory environment.

The regulatory modulation leads to the hypertrophy of the articular cartilage, which is characterized by physical changes, such as fraying, fibrillations with a rough surface, and the formation of osteophytes that become more prominent as the disease progresses [28]. We evaluated hypertrophy in our model by comparing the width of the right and left condyles and observed that the condyle wideness (in millimeters) was not different except when comparing the 30-day group treated with vitamin D during OA progression (sV + PsV) to the 30-day group supplemented only during the induction (sV + PnV). The comparison of these groups also revealed that vitamin D leads to the downregulation of TLR-4, MMP-3, and TNF-*α*. These findings are in accordance with a study indicating that vitamin D deficiency is greatly associated with the early stages of OA but absent in the advanced stages [4]. This could be explained by a recent report indicating that vitamin D precedes the TLR activation, with no effect observed when vitamin D is administrated at the same time or after TLR stimulation [32]. These data and our data suggest that vitamin D supplementation must be administrated before cartilage damage occurs.

Remarkably, we observed hypertrophy protection with low doses of vitamin D (4 IU) that was not observed in the same extends with higher doses (40–4000 IU). This lack of dose response might be due to the dual effect that vitamin D has on the different cells of the joint [28, 31]. In fact, it has been reported that administration of high doses of vitamin D induces side effects like hypercalcemia and hyperphosphatemia [15]. However, there is not an appropriate pharmacokinetic study that establishes the effects of vitamin D on the cartilage. Additionally, there are other factors that have to be taken into consideration, including the vitamin D-dependent induction of the expression of VEGF, growth factors [33], and MMPs [31, 33, 34]. These findings suggest that, in addition to its regulation of inflammation and damage, vitamin D could also contribute to factors highly associated with OA progression [28, 35].

In summary, our findings suggest that vitamin D may play a key role in the pathophysiology of OA. Additionally, the effects of vitamin D on OA may reflect the state of cell activation. Further studies are needed to examine the effects of vitamin D on the various molecules associated with the key degenerative or reparative processes within osteoarthritic cartilage. In addition, it is important to establish whether vitamin D supplementation in healthy subjects with serologically adequate levels of vitamin D yields a better response than supplementation in subjects with lower serum levels. However, it has been reported that healthy women with higher levels of vitamin D in serum had lower concentrations of TNF-*α* [36]. Moreover, a pharmacokinetic would help to understand the kinetics and effects of different doses of vitamin D.

## Supplementary Material

RNA was isolated from chondrocytes of knee cartilage samples and analyzed for the expression of TLR-4, IL-1*β*, TNF-*α*, and MMP-3 by a Real Time PCR system (Mod. 7500, Applied Biosystems, Carlsbad, CA, USA). The amounts of specific mRNA in the samples were calculated by the ΔΔCT method and the results were expressed as the relative expression level data (2-ΔΔC*т*). A correlation between the levels of relative expression of TLR-4 and IL-1*β*, TNF-*α*, and MMP-3 was performed using Pearson's correlation (Supplementary Figure 1).
4 IU (100 ng/kg/day) of vitamin D were administrated orally 3 days before surgery, and daily administrated until the last day of OA induction and/or progression (Figure 1). For the evaluation of vitamin D supplementation during OA progression, three experimental subgroups were included: (1) rats without vitamin supplementation during HIE + progression without vitamin supplementation (nV+PnV), (2) rats supplemented with the vitamin during HIE + progression without vitamin supplementation of vitamin (sV+PnV) and (3) rats supplemented with the vitamin during HIE + progression with vitamin supplementation (sV+PsV). The hypertrophy was evaluated measuring the wideness of the condyles using images from a stereoscopy microscope (Leica EZ4D. Bensheim, Alemania) and the software Gimp (GNU Image Manipulation Program). (Supplementary Figure 2).

## Figures and Tables

**Table 1:** Probes used for genes expression.

Gene	Sense	Antisense
TLR1	5′-TACCCTGAACAACGTGGACA-3′	5′-ATCGACAAAGCCCTCAGAGA-3′
TLR2	5′-GGAGACTCTGGAAGCAGGTG-3′	5′-CGCCTAAGAGCAGGATCAAC-3′
TLR4	5′-CCAGAGCCGTTGGTGTATCT-3′	5′-TCAAGGCTTTTCCATCCAAC-3′
TLR6	5′-GTCTCCCCACTTCATCCAGA-3′	5′-CCCACGTTTACCCTTCTCAA-3′
TNF*α*	5′-AAACTCGAGTGACAAGCCCG-3′	5′-GCAGCCTTGTCCCTTGAAGA-3′
IL1*β*	5′-CACCTCTCAAGCAGAGCACAG-3′	5′-GGGTTCCATGGTGAAGTCAAC-3′
IL6	5′-TCCTACCCCAACTTCCAATGCTC-3′	5′-TTGGATGGTCTTGGTCCTTAGCC-3′
MMP-3	5′-AGACAGGCACTTTTGGCG-3′	5′-CTTCATGACCTCGGATAGCC-3′
MMP-9	5′-TCGAATCACGGAGGAAGC-3′	5′-CCTAGCCCCAACTTATCCAG-3′
MMP-13	5′-CAAGGACCCTGGAGCCCTGA-3′	5′-TGAGGGTGCAGACGCCAGA-3′
GAPDH	5′-TCCTACCCCCAATGTATCCG-3′	5′-GGTGGAAGAATGGGAGTTGC-3′

## Abbreviations

OA: Osteoarthritis
HIE: High-impact exercise
TLRs: Toll-like receptors
CTL: Control
IL-1*β*: Interleukin-1*β*
IL-6: Interleukin-6
TNF*α*: Tumor necrosis factor alpha
MMP: Matrix metalloproteinase
DAMPs: Damage-associated molecular patterns
IU: International units
1, 25(OH)_2_D_3_: Vitamin D
14 HALO: 14 hours after light on
qPCR: Quantitative real-time polymerase chain reaction
HE: Hematoxylin and eosin
sV: Rats supplemented with the vitamin
nV: Rats without vitamin supplementation
nV + PnV: Rats without vitamin supplementation during HIE + progression without vitamin supplementation
sV + PnV: Rats supplemented with the vitamin during HIE + progression without vitamin supplementation
sV + PsV: Rats supplemented with the vitamin during HIE + progression with vitamin supplementation
ANOVA: Analysis of variance
GAPDH: Glyceraldehyde 3-phosphate dehydrogenase
C_T_: Cycle threshold
SEM: Standard error of the mean
NOD: Nucleotide oligomerization domain-like receptors.

## References

[1] Sadeghi K, Wessner B, Laggner U (2006). Vitamin D3 down-regulates monocyte TLR expression and triggers hyporesponsiveness to pathogen-associated molecular patterns. *European Journal of Immunology*.

[2] Haroon M, Bond U, Quillinan N, Phelan MJ, Regan MJ (2011). The prevalence of vitamin D deficiency in consecutive new patients seen over a 6-month period in general rheumatology clinics. *Clinical Rheumatology*.

[3] Bischoff-Ferrari HA, Zhang Y, Kiel DP, Felson DT (2005). Positive association between serum 25-hydroxyvitamin D level and bone density in osteoarthritis. *Arthritis & Rheumatism*.

[4] Heidari B, Heidari P, Hajian-Tilaki K (2011). Association between serum vitamin D deficiency and knee osteoarthritis. *International Orthopaedics*.

[5] Chaganti RK, Parimi N, Cawthon P, Dam TL, Nevitt MC, Lane NE (2010). Association of 25-hydroxyvitamin D with prevalent osteoarthritis of the hip in elderly men: the osteoporotic fractures in men study. *Arthritis and Rheumatism*.

[6] McAlindon TE, Felson DT, Zhang Y (1996). Relation of dietary intake and serum levels of vitamin D to progression of osteoarthritis of the knee among participants in the framingham study. *Annals of Internal Medicine*.

[7] Ding C, Cicuttini F, Parameswaran V, Burgess J, Quinn S, Jones G (2009). Serum levels of vitamin D, sunlight exposure, and knee cartilage loss in older adults: the Tasmanian older adult cohort study. *Arthritis and Rheumatism*.

[8] Felson DT, Niu J, Clancy M (2007). Low levels of vitamin D and worsening of knee osteoarthritis: results of two longitudinal studies. *Arthritis and Rheumatism*.

[9] Chlebowski RT, Johnson KC, Lane D (2011). 25-Hydroxyvitamin D concentration, vitamin D intake and joint symptoms in postmenopausal women. *Maturitas*.

[10] Muraki S, Dennison E, Jameson K (2011). Association of vitamin D status with knee pain and radiographic knee osteoarthritis. *Osteoarthritis and Cartilage*.

[11] Jefferies D, Farquharson C, Thomson J (2002). Differences in metabolic parameters and gene expression related to osteochondrosis/osteoarthrosis in pigs fed 25-hydroxyvitamin D3. *Veterinary Research*.

[12] Lane NE, Gore LR, Cummings SR (1999). Serum vitamin D levels and incident changes of radiographic hip osteoarthritis: a longitudinal study. Study of osteoporotic fractures research group. *Arthritis Rheum*.

[13] DeMarco PJ, Constantinescu F, Carbone LD, Barrow KD, Nevitt MC (2005). Does vitamin D supplementation contribute to the modulation of osteoarthritis by bisphosphonates? Comment on the article by Carbone et al. *Arthritis and Rheumatism*.

[14] Abbud K, Kouri J (2000). A novel rat osteoarthrosis model to assess apoptosis and matrix degradation. *Pathology Research and Practice*.

[15] Tsuruoka S, Nishiki K, Sugimoto K, Fujimura A (2002). Time of day improves efficacy and reduces adverse reactions of vitamin D3 in 5/6 nephrectomized rat. *Life Sciences*.

[16] Mills KHG, Dunne A (2009). Immune modulation: IL-1, master mediator or initiator of inflammation. *Nature Medicine*.

[17] Bobacz K, Sunk IG, Hofstaetter JG (2007). Toll-like receptors and chondrocytes: the lipopolysaccharide-induced decrease in cartilage matrix synthesis is dependent on the presence of toll-like receptor 4 and antagonized by bone morphogenetic protein 7. *Arthritis and Rheumatism*.

[18] Su SL, Tsai CD, Lee CH, Salter DM, Lee HS (2005). Expression and regulation of Toll-like receptor 2 by IL-1*β* and fibronectin fragments in human articular chondrocytes. *Osteoarthritis and Cartilage*.

[19] Kim HA, Cho ML, Choi HY (2006). The catabolic pathway mediated by Toll-like receptors in human osteoarthritic chondrocytes. *Arthritis and Rheumatism*.

[20] Liu-Bryan R, Pritzker K, Firestein GS, Terkeltaub R (2005). TLR2 signaling in chondrocytes drives calcium pyrophosphate dihydrate and monosodium urate crystal-induced nitric oxide generation. *Journal of Immunology*.

[21] Campo GM, Avenoso A, Campo S, D’Ascola A, Nastasi G, Calatroni A (2010). Small hyaluronan oligosaccharides induce inflammation by engaging both toll-like-4 and CD44 receptors in human chondrocytes. *Biochemical Pharmacology*.

[22] Khoo AL, Chai LYA, Koenen HJPM (2011). Regulation of cytokine responses by seasonality of vitamin D status in healthy individuals. *Clinical and Experimental Immunology*.

[23] Adams JS, Ren S, Liu PT (2009). Vitamin D-directed rheostatic regulation of monocyte antibacterial responses. *Journal of Immunology*.

[24] Liu PT, Stenger S, Li H (2006). Toll-like receptor triggering of a vitamin D-mediated human antimicrobial response. *Science*.

[25] Lu X, Farmer P, Rubin J, Nanes MS (2004). Integration of the Nf*κ*B p65 subunit into the vitamin D receptor transcriptional complex: identification of p65 domains that inhibit 1,25-dihydroxyvitamin D3-stimulated transcription. *Journal of Cellular Biochemistry*.

[26] Farmer PK, He X, Schmitz ML (2000). Inhibitory effect of NF-kappaB on 1,25-dihydroxyvitamin D(3) and retinoid X receptor function. *American Journal of Physiology. Endocrinology and Metabolism*.

[27] Wu S, Liao AP, Xia Y (2010). Vitamin D receptor negatively regulates bacterial-stimulated NF-*κ*B activity in intestine. *American Journal of Pathology*.

[28] Tetlow LC, Woolley DE (2001). Expression of vitamin D receptors and matrix metalloproteinases in osteoarthritic cartilage and human articular chondrocytes in vitro. *Osteoarthritis and Cartilage*.

[29] Lee BNR, Kim TH, Jun JB (2011). Upregulation of interleukin-1*β* production by 1,25-Dihydroxyvitamin D3 in activated human macrophages. *Molecular Biology Reports*.

[30] Do JE, Kwon SY, Park S, Lee ES (2008). Effects of vitamin D on expression of Toll-like receptors of monocytes from patients with Behçet’s disease. *Rheumatology*.

[31] Tetlow LC, Wooley DE (1999). The effects of 1*α*,25-dihydroxyvitamin D3 on matrix metalloproteinase and prostaglandin E2 production by cells of the rheumatoid lesion. *Arthritis Research*.

[32] Gambhir V, Kim J, Siddiqui S (2011). Influence of 1,25-dihydroxy vitamin D3 on TLR4-induced activation of antigen presenting cells is dependent on the order of receptor engagement. *Immunobiology*.

[33] Lin R, Amizuka N, Sasaki T (2002). 1*α*,25-dihydroxyvitamin D3 promotes vascularization of the chondro-osseous junction by stimulating expression of vascular endothelial growth factor and matrix metalloproteinase 9. *Journal of Bone and Mineral Research*.

[34] Tetlow LC, Woolley DE (1999). Effects of 1*α*,25dihydroxyvitaminD3 on matrix metalloproteinase expression by rheumatoid synovial cells and articular chondrocytes in vitro. *Annals of the New York Academy of Sciences*.

[35] Haywood L, McWilliams DF, Pearson CI (2003). Inflammation and angiogenesis in osteoarthritis. *Arthritis and Rheumatism*.

[36] Peterson CA, Heffernan ME (2008). Serum tumor necrosis factor-alpha concentrations are negatively correlated with serum 25(OH)D concentrations in healthy women. *Journal of Inflammation*.

