# Supplementary material for: Effects of Vitamin D Supplementation during the Induction and Progression of Osteoarthritis in a Rat Model
Source: Evid Based Complement Alternat Med. 2012 Oct 14;2012:156563. doi: 10.1155/2012/156563 (PMC3479853; doi:10.1155/2012/156563)
Supplement: Supplementary file 1 — RNA was isolated from chondrocytes of knee cartilage samples and analyzed for the expression of TLR-4, IL-1β, TNF-α, and MMP-3 by a Real Time PCR system (Mod. 7500, Applied Biosystems, Carlsbad, CA, USA). The amounts of specific mRNA in the samples were calculated by the ΔΔCT method and the results were expressed as the relative expression level data (2-ΔΔCт). A correlation between the levels of relative expression of TLR-4 and IL-1β, TNF-α, and MMP-3 was performed using Pearson's correlation (Supplementary Figure 1). 4 IU (100 ng/kg/day) of vitamin D were administrated orally 3 days before surgery, and daily administrated until the last day of OA induction and/or progression (Figure 1). For the evaluation of vitamin D supplementation during OA progression, three experimental subgroups were included: (1) rats without vitamin supplementation during HIE + progression without vitamin supplementation (nV+PnV), (2) rats supplemented with the vitamin during HIE + progression without vitamin supplementation of vitamin (sV+PnV) and (3) rats supplemented with the vitamin during HIE + progression with vitamin supplementation (sV+PsV). The hypertrophy was evaluated measuring the wideness of the condyles using images from a stereoscopy microscope (Leica EZ4D. Bensheim, Alemania) and the software Gimp (GNU Image Manipulation Program). (Supplementary Figure 2). [file 156563.f1.docx]

|  |
| --- |
| Supplementary figure 1. TLR-4 expression is correlated with expression of the MMP-3, TNF-α, and IL-1β in OA. A. The correlation between TLR-4 and MMP-3 expression has an R^2^= 0.7692. B. The correlation between TLR-4 and TNF-α expression has an R^2^= 0.5945. C. The correlation between TLR-4 and IL-1β expression has an R^2^=0.3954. The values of *p=0.0169 and **p=0.0095 were determined using Pearson’s correlation analysis. |

|  |
| --- |
| Supplementary figure 2. Vitamin D reduces the hypertrophy induced by OA progression. Representative images of femoral condyles with lesions from rats treated with vitamin D for 3, 10, or 20 days of OA induction (sV), and rats with OA progression with or without 4 UI of vitamin D supplementation on days 23, 30, and 40: sV+PsV, sV+PnV, and nV+PnV are shown. The scale bar= 1 mm at 12.5 X magnification. |
